# Plasma Exosomal Brain-Derived Neurotrophic Factor Correlated with the Postural Instability and Gait Disturbance–Related Motor Symptoms in Patients with Parkinson’s Disease

**DOI:** 10.3390/diagnostics10090684

**Published:** 2020-09-11

**Authors:** Chen Chih Chung, Pai Hao Huang, Lung Chan, Jia-Hung Chen, Li-Nien Chien, Chien Tai Hong

**Affiliations:** 1 Department of Neurology, Shuang Ho Hospital, Taipei Medical University, Zhongzheng Rd, Zhonghe District, New Taipei City 23561, Taiwan; 10670@s.tmu.edu.tw (C.C.C.); cjustinmd@tmu.edu.tw (L.C.); gary.320@hotmail.com (J.-H.C.); 2 Department of Neurology, School of Medicine, College of Medicine, Taipei Medical University, Taipei 11031, Taiwan; 3 Graduate Institute of Biomedical Informatics, Taipei Medical University, Taipei 11031, Taiwan; 4 Department of Neurology, Cathay General Hospital, Taipei 106, Taiwan; Paihao.huang@gmail.com; 5 School of Health Care Administration, College of Management, Taipei Medical University, 250 Wuxing Street, Taipei 11031, Taiwan

**Keywords:** exosomes, Parkinson’s disease, brain-derived neurotrophic factor, biomarker, postural instability and gait disturbance

## Abstract

Brain-derived neurotrophic factor (BDNF) is an essential neurotrophin, responsible for neuronal development, function, and survival. Assessments of peripheral blood BDNF in patients with Parkinson’s disease (PD) previously yielded inconsistent results. Plasma exosomes can carry BDNF, so this study investigated the role of plasma exosomal BDNF level as a biomarker of PD. A total of 114 patients with mild to moderate PD and 42 non-PD controls were recruited, and their clinical presentations were evaluated. Plasma exosomes were isolated with exoEasy Maxi Kits, and enzyme-linked immunosorbent assay was used to assess plasma exosomal BDNF levels. Statistical analysis was performed using SPSS version 19.0, and findings were considered significant at *p* < 0.05. The analysis revealed no significant differences in plasma exosomal BDNF levels between patients with PD and controls. Patients with PD with low plasma exosomal BDNF levels (in the lowest quartile) exhibited a significant association with daily activity dysfunction but not with cognition/mood or overall motor symptoms as assessed using the Unified Parkinson’s Disease Rating Scale (UPDRS). Investigation of UPDRS part III subitems revealed that low plasma exosomal BDNF level was significantly associated with increased motor severity of postural instability and gait disturbance (PIGD)-associated symptoms (rising from a chair, gait, and postural stability) after adjustment for age and sex. In conclusion, although plasma exosomal BDNF level could not distinguish patients with PD from controls, the association with PIGD symptoms in patients with PD may indicate its potential role as a biomarker. Follow-up studies should investigate the association between plasma exosomal BDNF levels and changes in clinical symptoms.

## 1. Introduction

Parkinson’s disease (PD) is among one of the most common neurodegenerative diseases, second only to Alzheimer’s disease (AD) in epidemiology [1]. The underlying cause of PD is the degeneration of dopaminergic neurons in the substantia nigra due to multifactorial cellular stress, including oxidative stress, protein misfolding, neuroinflammation, and lysosomal dysfunction [2]. Neurotrophins are endogenous proteins responsible for the development, function, and survival of neurons [3]. The expression of neurotrophins in the brain is downregulated in postmortem patients with PD [4,5], and the supplements of neurotrophin are found to be neuroprotective in animal studies of PD and clinical trials [6].

Brain-derived neurotrophic factor (BDNF) is one of the most abundant neurotrophins and is expressed in multiple brain areas, including the hippocampus, frontal cortex, midbrain, amygdala, hypothalamus, striatum, pons, and medulla oblongata. It modulates neuronal differentiation, development, survival, synaptogenesis, and synaptic plasticity [7]. BDNF deficiencies have been noted in several neurological and psychological diseases [8]. Aside from neurons, several somatic cells also express BDNF, and physical activity has been demonstrated to stimulate neuronal and nonneuronal-origin BDNF [9]. Blood BDNF has also been investigated for its possible role as a biomarker for PD. Blood BDNF was assumed to be lower in patients with PD than in controls because of the nature of neurodegenerative diseases; however, prior research found heterogenicity to be remarkable [10]. Moreover, the blood BDNF level is dynamic and is affected by numerous environmental factors [7]. The half-life of blood BDNF is short, which is disadvantageous for a biomarker [11].

Extracellular vesicles (EVs) are small cargoes secreted from the cells and carrying abundant proteins and nucleotides [12]. Three different significant subtypes of EVs were distinguished based on the dimension, origin, and markers. Apoptotic vesicles, >1000 nm in dimension, originate by the breakdown of apoptotic cells and contain histones; microvesicles, 100–1000 nm in dimension, originate from blebbing of the cellular plasma membrane and exosomes, <100 nm in dimension, derived from the fusion of multivesicular bodies with the cell membrane and positive with tetraspanins, such as CD9, CD63, and CD81 [13]. In the last decade, exosome has attracted the most research attention among all the EVs. Because of their lipid bilayer outer membrane, exosomes can cross the blood–brain barrier (BBB) and remain stable in the blood for long periods [14]. This high structural stability prevents the degradation of circulating biomarkers and reflects steady conditions in the peripheral blood. The inner contents of plasma exosomes have been found to provide evidence of disease pathogenesis, such as an association between AD and insulin resistance [15,16]. Besides, exosomes carry intracellular proteins, such as α-synuclein, β-amyloid, and tau, which may reflect cellular pathology if the engulfment of proteins into exosome is cytoplasm mimicking [17].

This study investigated the associations between PD with plasma exosomal BDNF level and between plasma exosomal BDNF level with specific clinical manifestations of PD.

## 2. Materials and Methods

### 2.1. Study Participants

A total of 156 patients (114 patients with PD and 42 controls) were enrolled. Diagnosis of PD was based on the UK Parkinson’s Disease Society Brain Bank diagnostic criteria [18]. Only patients with mild to moderate PD, defined as stages I to III according to the Hoehn and Yahr scale, were included in the PD group. The controls were free from known neurodegenerative, psychiatric, and major systemic diseases (malignant neoplasm and chronic kidney disease), and were regularly followed up in outpatient clinics for chronic conditions (hypertension, diabetes, or hyperlipidemia). In the control group, patients with Mini-Mental State Examination (MMSE) scores of less than 26 were excluded. The Joint Institutional Review Board of Taipei Medical University approved this study (N201609017 on 25 November 2016 and N201801043 on 23 February 2018).

### 2.2. Clinical Assessments

All participants were interviewed to obtain baseline demographic data. The cognitive functions of all study participants were investigated by trained nurses using the Taiwanese versions of the MMSE and Montreal Cognitive Assessment (MoCA). All patients with PD were evaluated using parts I, II, and III of the Unified Parkinson’s Disease Rating Scale (UPDRS) during an outpatient visit. The time between the most recent dose of anti-PD medication and the assessment of UPDRS part III was not recorded; patients with PD were assumed to be in their “on” period.

### 2.3. Plasma Exosomes Separation and Validation

Plasma exosomes separation was performed mainly following the International Society of Extracellular Vesicles guidelines [19]. In brief, venous blood samples were collected from all study participants. Whole blood was centrifuged at 13,000× *g* for 20 min to isolate the plasma. Next, 1 mL of plasma from each participant was subjected to an exoEasy Maxi Kit (Qiagen, Valencia, CA, USA) for exosome isolation according to the manufacturer’s instructions. The last step of isolation was the elution of the exosomes from the column. In most cases, 400 μL of elution was obtained.

Detection of the presence of tetraspanins CD9 and CD63, and the tumor susceptibility gene 101 (TSG101) confirmed the isolation of exosomes. The size distribution of isolated exosomes was determined through nanoparticle tracking. For Western blot analysis, anti-CD9 antibody (ab92726, Abcam, Cambridge, UK), anti-CD63 antibody (ab59479, Abcam, Cambridge, UK), anti-TSG101 antibody (GTX118736, GeneTex, CA, USA), and anti-heat shock protein (HSP) 70 (NBP1-77456, Novus Bio, CO, USA) were used at a 1:1000 dilution. Nanoparticle tracking was performed using a NanoSight NS300 (Malvern Panalytical, Malvern, UK) under the manufacturer’s instructions.

### 2.4. Assessment of BDNF

Enzyme-linked immunosorbent assay (ELISA; ab212166, Abcam, Cambridge, UK) was used according to the manufacturer’s instructions to assess the exosomal BDNF level. Each sample was assessed in duplicate, and the concentration of BDNF was compared with the standard sample provided.

### 2.5. Statistical Analysis

All statistical analyses were performed using SPSS software version 19 for Windows 10 (SPSS Inc., Chicago, IL, USA). A chi-square test was performed to compare the gender distribution between patients with PD and controls, and the severity of motor symptoms based on UPDRS between PD patients with low and optimal exosomal BDNF. A nonparametric Mann–Whitney U test was performed to compare the plasma exosomal levels of BDNF and other continuous variables between PD and controls patients. Multivariable logistic regression was applied to investigate the association between plasma exosomal BDNF level and motor severity in patients with PD, after adjustment for age and sex. One-way analysis of variance with Dunnett’s post hoc analysis was used for multiple comparisons. A *p*-value of < 0.05 was considered statistically significant.

## 3. Results

**Figure 1:**
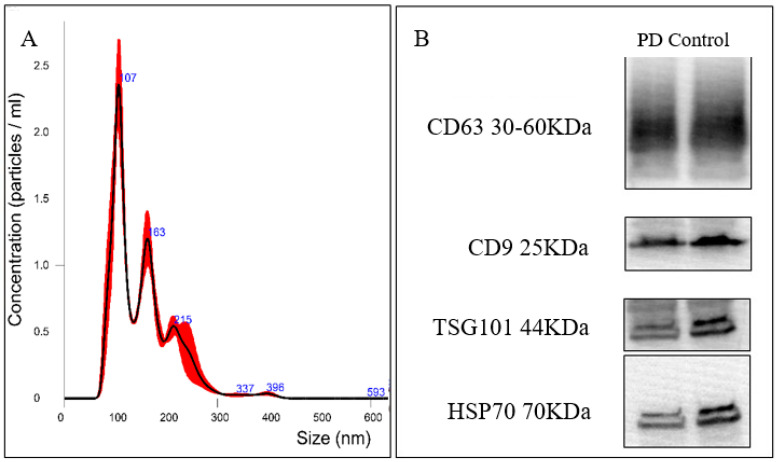
Characterization of isolated plasma exosomes. (**A**) Nanoparticle tracking analysis of the size distribution of isolated exosomes. (**B**) Representative Western blot images of the differential expression of exosomal markers CD63, CD9, and TSG101 in exosomes isolated from controls and Parkinson’s disease (PD) patients. HSP70 served as the loading control of exosomes.

The isolated plasma exosomes were validated through nanoparticle tracking. In both controls and PD patients, the harvested exosomes’ size was approximately 35–150 nm (Figure 1A). The exosomes were also subjected to protein quantification to detect tetraspanins CD9 and CD63, and TSG101. In representative Western blot analysis, the CD9, CD63, and TSG101 immunostaining intensities for exosomes from both controls and PD patients were strongly positive (Figure 1B).

**Figure 2:**
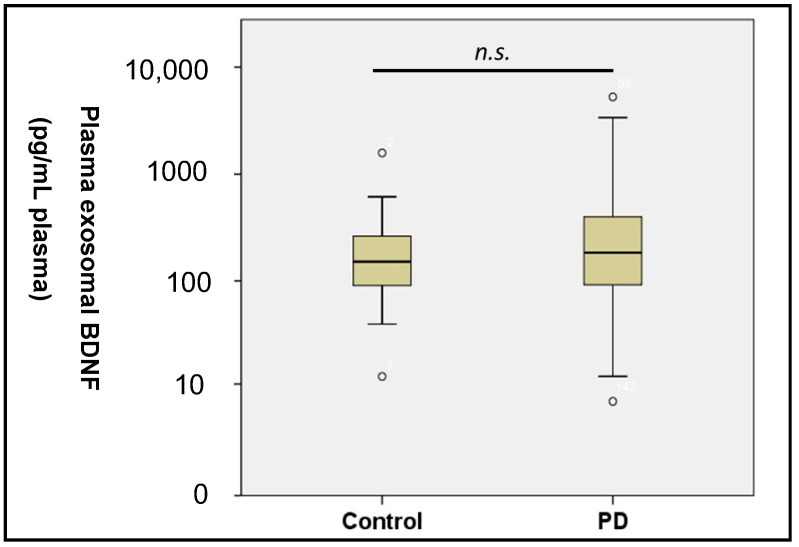
Plasma exosomal brain-derived neurotrophic factors (BDNF) levels among controls and patients with Parkinson’s disease (PD). Data are presented as the median, first quartile, third quartile, and variation. *n.s.*, nonsignificant.

The baseline data are presented in Table 1. No differences in age or gender were observed between patients with PD and controls. Because the control group excluded people with MMSE scores less than 26, significant cognitive function differences between PD and controls were identified from MMSE and MoCA scores. The mean PD duration was 2.70 ± 2.45 years, and the mean UPDRS scores in parts I, II, and III were 2.44 ± 1.98, 8.06 ± 5.80, and 22.89 ± 10.00 years, respectively. Plasma exosomal BDNF levels were not significantly different between patients with PD and controls (PD: 374.6 ± 683.6 pg/mL plasma; control: 213.6 ± 252.0 pg/mL plasma, *p* = 0.24) (Figure 2).

**Figure 3:**
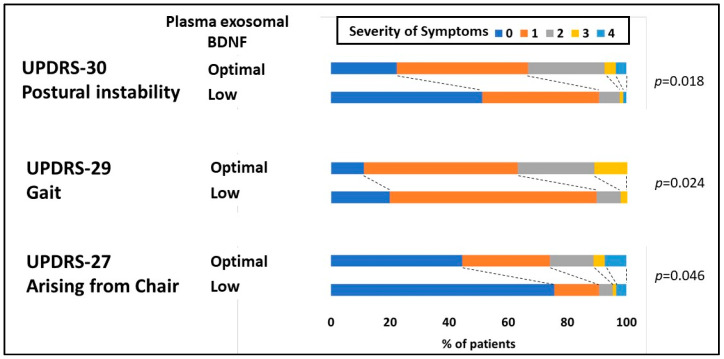
Distribution of the severity of PIGD-related motor symptoms (arising from a chair, gait, and postural stability) within the Unified Parkinson’s Disease Rating Scale (UPDRS) subitem in patients with PD between those with low plasma exosomal brain-derived neurotrophic factors (BDNF) (Q1) and optimal plasma exosomal BDNF (Q2–4) levels.

Patients with PD were categorized into four groups based on the quartile of their plasma exosomal BDNF level. Disease severity was assessed using UPDRS parts I, II, and III. Compared with patients in the other groups, patients with PD who had a low plasma EV BDNF level (Q1) performed significantly worse in part II (daily activity) but not in part I (cognition and mood) or part III (motor symptoms as evaluated by an examiner) (Table 2). However, when part III was subdivided into individual items, patients with PD in the group with the lowest plasma exosomal BDNF level (Q1) performed significantly worse on items 27 (arising from a chair), 29 (gait), and 30 (postural stability) (Supplementary Table S1). Furthermore, after the group with a low plasma exosomal BDNF level (Q1) was distinguished from the groups with optimal plasma exosomal BDNF levels (Q2–4), the severity of postural instability and gait disturbance (PIGD)-associated motor symptoms were found to be significantly worse in the low plasma exosomal BDNF level group (Figure 3). After adjustment for age and sex, low plasma exosomal BDNF level was observed to significantly contribute to a 0.46 ± 0.22 point increase on item 27 (arising from a chair, *p* = 0.036), 0.36 ± 0.15 point increase on item 29 (gait, *p* = 0.014), and 0.55 ± 0.18 point increase on item 30 (postural stability, *p* = 0.003) (Table 3).

## 4. Discussion

The study demonstrates that PD patients are indistinguishable from controls concerning plasma exosomal BDNF level; however, plasma exosomal BDNF level may be a segregating biomarker for the PIGD subtype of PD. Compared with PD patients with optimal plasma exosomal BDNF, patients with low plasma exosomal BDNF levels in this study were likely to exhibit more significant dysfunction in PIGD-associated motor symptoms, which affect the quality of life and are often unmitigated by medical treatment [20]. Considering that PIGD is an unfavorable subtype of PD that may transform from benign subtypes of PD [21], plasma exosomal BDNF level may potentially predict and monitor disease progression in PD.

Blood BDNF, found in either plasma or serum, was investigated as a biomarker for numerous neurological and psychological disorders. Plasma BDNF is optimal for biomarker identification because the activation of platelets induces BDNF expression, which would otherwise lead to bias in the amount of BDNF detected in serum [22]. A correlation between plasma BDNF and brain BDNF expression has been confirmed [23], although the ability of BDNF to cross the BBB remains debatable due to inconsistent results [24,25,26]. BDNF is presumed to be responsible for the survival and repair of neurons, and low plasma BDNF has been thought to be related to neurodegenerative diseases. However, plasma BDNF was found to be elevated, reduced, or identical in patients with PD compared with controls, as demonstrated by a recent meta-analysis conducted by Jiang et al. [10]. This inconsistency was also noted by research on the association between BDNF and AD [27]. The compensatory response of elevated BDNF expression in neurons in the early stages of the neurodegenerative disease was one possible explanation for elevated BDNF in these patients [28]. Because of the short half-life (less than 60 min of BDNF in plasma [24,29]), the measurement of plasma BDNF is affected by lifestyle factors, such as exercise, diet, and smoking habits. Rather than measuring plasma BDNF, this study took plasma exosomes as a source of BDNF. Exosomes can cross the BBB without compromising integrity. The possible mechanisms for the exosomes to cross the BBB include direct translocation into capillaries or draining venules via the BBB, passing through interstitial fluid into the cerebrospinal fluid and then in the venous system via the arachnoid granulations, transportation into the perinasal lymphatics and then into the venous system, and the modulation of the endothelial cell on the BBB by peripheral exosomes for the adjustment of its permeability [30,31]. The circulating half-life of exosomes in the blood is more protracted, thus avoiding short-term event-related BDNF surges. Plasma exosomal BDNF level has been assessed concerning healthy aging [32], but not in patients with neurodegenerative diseases. The present study demonstrated that an abundance of BDNF in plasma exosomes was detectable by conventional ELISA. However, plasma exosomal BDNF levels could not distinguish patients with PD from controls. Levodopa may transiently increase plasma BDNF [33], and nearly all patients with PD in our cohort were prescribed levodopa for symptom management. As mentioned above, the compensation in BDNF expression in the early stages of the disease was also possible. Because BDNF is expressed in multiple brain areas and is nonspecific to PD, the application of plasma exosomal BDNF level as a PD diagnostic biomarker may be impractical.

Nevertheless, the present study discovered an association between plasma exosomal BDNF levels and the severity of PIGD-associated motor symptoms in PD patients. PD is a heterogeneous disease with different motor subtypes, including tremor-predominant PD, akinetic-rigidity–predominant PD, and PIGD. Distinct from other subtypes, PIGD is characterized by rapid progression, comorbidity with cognitive dysfunction, and motor symptoms that are unresponsive to levodopa treatment [20]. Several brain conditions are associated with PIGD, including gray matter atrophy [34], basal ganglia dysfunction [35], white matter lesions [36], and cerebral microbleeds [37], indicating a multifaceted brain pathology. Motor learning is crucial for the control of postural stability and gait. PD patients, especially the PIGD subgroup, have difficulty acquiring movement schema to learn tasks. Neuroanatomically, the motor and premotor cortex are responsible for motor learning [38], expressing BDNF. The deficiency of BDNF has been found to affect the motor cortex and motor performance more than it does the hippocampus [39]. In the rat model of ischemic stroke, antisense BDNF oligonucleotide administration negated rehabilitation’s therapeutic effect on the recovery of skilled reaching [40]. Besides, the carriers of Val66Met polymorphism of BDNF, a disadvantaged variant in the function of BDNF, were worse in the locomotor adaptation after stroke [41]. The proBDNF, which is functionally opposite to BDNF, increased the elders’ exosomes with walking speed decline [32]. This evidence suggested that the correlation between exosomal BDNF with the PIGD subtype of PD may reflect from the association of BDNF with a deficit in motor and premotor cortex-related motor learning. Another alternative for the correlation between exosomal BDNF with PIGD was the higher risk of falling-related limiting physical activity for these subgroups of PD patients. The exercise was found to increase the brain expression of BDNF [42], which indicates another indirect relationship between low plasma exosomal BDNF level and the PIGD subtype.

One strength of the present study is that it is the first to investigate plasma exosomal BDNF levels in PD patients. The findings prove that plasma exosomal BNDF is measurable by conventional ELISA and associated with PIGD-related motor symptoms. There is currently no candidate blood biomarker for subtyping patients with PD. However, identifying patients with PIGD who are unresponsive to levodopa, at high risk of disease progression, or exhibit brain pathology with higher comorbidity than other patients with PD is necessary. These patients require unique management and should not be treated the same way as other PD patients, especially deep brain stimulation [43]. Besides, patients with PD of non-PIGD subtypes may convert to the PIGD subtype as the disease progresses [21]; thus, a reliable blood biomarker is required to make early prediction possible.

The present study has some limitations. First, total plasma exosomes were used instead of neuron-derived exosomes. Because BDNF expression was not limited in brain tissue, and somatic cells may have also secreted exosomes containing BDNF. However, exosomes cross the BBB bidirectionally. The present study measured the total plasma exosomal BDNF, which originated from both CNS and somatic tissues, reflecting the combinations of the BDNF secretion capability of CNS and the feedback from the somatic tissues. Second, this study did not measure the genetic polymorphism of BDNF, the isoforms of BDNF in plasma exosome, and other downstream proteins responsible for the action of BDNF. These factors also affect the BDNF downstream signaling pathway [44,45]. Third, the control participants were not typical healthy controls as in other studies, but age was controlled, and the controls were regularly followed up in the outpatient clinic for their underlying diseases, including hypertension, diabetes, and hyperlipidemia. Although they did not have subjective memory decline, some may have fallen into the category of mild cognitive impairment, which would indicate comorbidity with vascular or AD pathologies in the control group. The lack of the investigation of the correlation between plasma BDNF with the plasma exosomal BDNF was a shortcoming as well. Lastly, it was a cross-sectional study, and a longitudinal cohort to investigate the predicting accuracy of plasma exosomal BDNF on the conversion to PIGD subtype of PD is warranted.

In conclusion, patients with PD exhibited no differences in plasma exosomal BDNF levels compared with controls. Lower plasma exosomal BDNF levels were associated with more severe axial motor symptoms in patients with PD. We recommend that further longitudinal studies investigate the correlation between plasma exosomal BDNF levels in PD and future disease progression patients.

## Figures and Tables

**Table 1:** Demographic data of patients with Parkinson’s disease (PD) and controls.

	Controls	PD	*p* Value
Number of patients	42	114	-
Age (years)	67.02 ± 7.00	69.67 ± 8.44	0.12
Female patients	13	52	0.14
Disease duration (years)	-	2.70 ± 2.45	-
MMSE	28.50 ± 1.21	24.88 ± 5.03	<0.001
MoCA	24.05 ± 2.93	20.41 ± 6.08	0.001
UPDRS-I		2.44 ± 1.98	-
UPDRS-II		8.06 ± 5.80	-
UPDRS-III		22.89 ± 10.00	-

MMSE, Mini-Mental State Examination; MoCA, Montreal Cognitive Assessment; UPDRS, Unified Parkinson’s Disease Rating Scale.

**Table 2:** Unified Parkinson’s Disease Rating Scale (UPDRS) scores of patients with PD categorized into four groups based on the quartile (Q1 to Q4, low to high) for the plasma exosomal brain-derived neurotrophic factors (BDNF) level.

	Plasma Exosomal BDNF at Q1	Plasma Exosomal BDNF at Q2	Plasma Exosomal BDNF at Q3	Plasma Exosomal BDNF at Q4	*p* for Trend
UPDRS I	2.96 ± 2.70	2.17 ± 1.51	2.24 ± 1.70	2.43 ± 1.89	0.447
UPDRS II	10.59 ± 6.78	7.45 ± 5.00	8.03 ± 6.19	6.29 ± 4.46	0.042
UPDRS III	26.04 ± 11.14	21.24 ± 9.73	21.52 ± 10.15	23.00 ± 8.71	0.263

**Table 3:** Correlations between low plasma exosomal BDNF levels (Q1) and PIGD-related subitems in UPDRS part III after adjustment for age and sex.

	B	SE.	*p* Value
Arising from the chair	0.460	0.217	0.036
Gait	0.364	0.146	0.014
Postural stability	0.546	0.180	0.003

B, coefficient; SE, standardized error.

## Supplementary Materials

The following are available online at https://www.mdpi.com/2075-4418/10/9/684/s1, Table S1: The comparison of scores in subitem of UPDRS part III between PD patients with low to high EV BDNF levels.
