# Supplementary material for: Plasma Exosomal Brain-Derived Neurotrophic Factor Correlated with the Postural Instability and Gait Disturbance–Related Motor Symptoms in Patients with Parkinson’s Disease
Source: Diagnostics (Basel). 2020 Sep 11;10(9):684. doi: 10.3390/diagnostics10090684 (PMC7555255; doi:10.3390/diagnostics10090684)
Supplement: Supplementary file 1 [file diagnostics-10-00684-s001.pdf]

Supplementary Table S1. The comparison of scores in subitem of Unified Parkinson's Disease Rating Scale (UPDRS) part III between Parkinson's disease patients with low to high quartile (Q1 to Q4) exosomal brain-derived neurotrophic factor (BDNF) levels.

	Plasma exosomal BDNF at Q1	Plasma exosomal BDNF at Q2	Plasma exosomal BDNF at Q3	Plasma exosomal BDNF at Q4	*p* for trend
UPDRS-18	0.93 ±0.55	0.93 ±0.37	0.76 ±0.51	1.04 ±0.43	0.170
UPDRS-19	1.26 ±0.71	1.38 ±0.62	1.31 ±0.60	1.61 ±0.50	0.161
UPDRS-20	1.41 ±1.93	1.21 ±1.61	1.10 ±1.91	1.04 ±1.75	0.884
UPDRS-21	1.26 ±1.10	1.72 ±1.72	1.62 ±1.35	1.25 ±1.58	0.500
UPDRS-22	5.15 ±2.52	4.14 ±2.59	4.41 ±2.39	5.68 ±3.42	0.144
UPDRS-23	2.81 ±1.69	2.28 ±1.58	2.38 ±1.50	2.54 ±1.53	0.605
UPDRS-24	2.07 ±1.47	1.76 ±1.35	1.76 ±1.51	1.79 ±1.32	0.780
UPDRS-25	2.48 ±1.60	2.03 ±1.27	2.10 ±1.40	2.18 ±1.33	0.653
UPDRS-26	2.15 ±1.35	1.86 ±1.51	1.79 ±1.45	2.00 ±1.47	0.804
UPDRS-27	1.00 ±1.21	0.41 ±0.95	0.66 ±1.17	0.18 ±0.39*	**0.018**
UPDRS-28	1.03 ±0.44	0.97 ±0.50	0.86 ±0.64	0.96 ±0.50	0.667
UPDRS-29	1.37 ±0.84	0.93 ±0.59*	0.93 ±0.70*	0.93 ±0.54*	**0.038**
UPDRS-30	1.22 ±0.97	0.55 ±0.95*	0.69 ±0.71*	0.61 ±0.63*	**0.012**
UPDRS-31	1.48 ±0.58	1.07 ±0.70	1.14 ±0.64	1.21 ±0.69	0.104

Data were presented as mean ±standard deviation. *, $p < 0.05$ in the Dunnett's post-hoc analysis as comparison with the Q1 group and presented in bold style.
